# Oleanolic Acid Modulates DNA Damage Response to Camptothecin Increasing Cancer Cell Death

**DOI:** 10.3390/ijms252413475

**Published:** 2024-12-16

**Authors:** Giulio Mazzarotti, Maria Cuomo, Maria Carmen Ragosta, Andrea Russo, Margherita D’Angelo, Annamaria Medugno, Giuseppe Maria Napolitano, Carmelina Antonella Iannuzzi, Iris Maria Forte, Rosa Camerlingo, Sharon Burk, Francesco Errichiello, Luigi Frusciante, Martino Forino, Maria Rosaria Campitiello, Michelino De Laurentiis, Antonio Giordano, Luigi Alfano

**Affiliations:** 1 Department of Medical Biotechnologies, University of Siena, 53100 Siena, Italy; 2 Clinical and Translational Oncology Program, Scuola Superiore Meridionale (SSM, School of Advanced Studies), University of Naples Federico II, 80131 Naples, Italy; 3 Unit of Dietetics and Sports Medicine, Department of Experimental Medicine, Section of Human Physiology, Università degli Studi della Campania “Luigi Vanvitelli”, 80122 Naples, Italy; 4 Department of Breast and Thoracic Oncology, Istituto Nazionale Tumori-IRCCS-Fondazione G. Pascale, 80131 Naples, Italy; 5 Cell Biology and Biotherapy Unit, Istituto Nazionale Tumori-IRCCS-Fondazione G. Pascale, 80131 Naples, Italy; 6 Department of Agricultural Sciences, Grape and Wine Science Division, University of Napoli Federico II, 83100 Avellino, Italy; 7 Department of Obstetrics and Gynecology and Physiopathology of Human Reproduction, ASL Salerno, 84124 Salerno, Italy; 8 Sbarro Institute for Cancer Research and Molecular Medicine, Center for Biotechnology, College of Science and Technology, Temple University, BioLife Science Bldg. Suite 333, 1900 N 12th Street, Philadelphia, PA 19122, USA

**Keywords:** DNA damage response, homologous recombination, DNA repair, single-strand annealing, oleanolic acid, Aglianico grape pomace

## Abstract

Targeting DNA damage response (DDR) pathways represents one of the principal approaches in cancer therapy. However, defects in DDR mechanisms, exhibited by various tumors, can also promote tumor progression and resistance to therapy, negatively impacting patient survival. Therefore, identifying new molecules from natural extracts could provide a powerful source of novel compounds for cancer treatment strategies. In this context, we investigated the role of oleanolic acid (OA), identified in fermented Aglianico red grape pomace, in modulating the DDR in response to camptothecin (CPT), an inhibitor of topoisomerase I. Specifically, we found that OA can influence the choice of DNA repair pathway upon CPT treatment, shifting the repair process from homologous recombination gene conversion to single-strand annealing. Moreover, our data demonstrate that combining sub-lethal concentrations of OA with CPT enhances the efficacy of topoisomerase I inhibition compared to CPT alone. Overall, these findings highlight a new role for OA in the DDR, leading to a more mutagenic DNA repair pathway and increased sensitivity in the HeLa cancer cell line.

## 1. Introduction

Defects in the DDR are among the leading causes of genome instability and cancer development [1]. DNA double-strand breaks (DSBs) are particularly toxic to cells and are primarily repaired through two mechanisms: homologous recombination (HR) and non-homologous end joining (NHEJ) [2]. HR is an error-free repair process that primarily occurs during the S/G2 phases of the cell cycle. It begins with DNA end-resection, which generates long 3′-single-strand DNA. This single-strand DNA is crucial for strand invasion, a process mediated by RAD51, which facilitates the accurate copying of genetic information from sister chromatids [3]. Moreover, HR is not the only pathway that repairs DSBs, but it collaborates and competes with other DNA repair mechanisms; in particular, upon the generation of 3′-single-standed DNA tails resulting from DNA end-resection, non-conservative single-strand annealing (SSA) or alternative end-joining (Alt-EJ) can compete with gene conversion (GC) for DNA repair [4]. Conversely, NHEJ repairs DNA lesions through insertion or deletion, making it an error-prone repair mechanism. The choice between HR and NHEJ for DNA repair is an active research topic due to its implications in cancer progression and its potential as a therapeutic strategy for tumors [5,6]. Various proteins involved in the response to DSBs—including sensors, transducers, mediators, and effectors—are frequently mutated in tumor samples. This suggests that defects in DDR pathways significantly impact cancer biology [7]. The discovery of synthetic lethality through PARP1 inhibition in BRCA1/2-deficient tumors has ushered in a new era of personalized medicine in cancer therapy [8].

Many screenings have been initiated to identify new potential synthetic lethal interactions, aiming to expand cancer treatment approaches. With the rise in resistance mechanisms to standard-of-care cancer therapies and the recent advances in synthetic lethality, identifying new drugs for tumor therapy has become crucial.

In line with these objectives, we have launched a new research program in collaboration with the Department of Agricultural Sciences at the University of Naples Federico II to discover novel natural compounds for cancer therapy [9]. Herein, we describe the ability of an extract from Aglianico grape pomace (*Vitis vinifera* L. cv) to modulate the DDR in the context of camptothecin treatment. An NMR-based analysis of the pomace extract identified several compounds, with OA being one of the most abundant. OA has previously been recognized as a metabolite involved in modulating resistance to radiotherapy [10]. Here, we report that the combination of OA with CPT treatment increases the sensitivity in HeLa cells by directing DNA repair through non-conservative mechanisms. These findings support the potential role of OA in cancer therapy through its modulation of the DNA damage response.

## 2. Results

### 2.1. Fermented Red Grape Pomace Affects Hela Cell Viability and DNA Repair in Response to CPT

**Figure 1:**
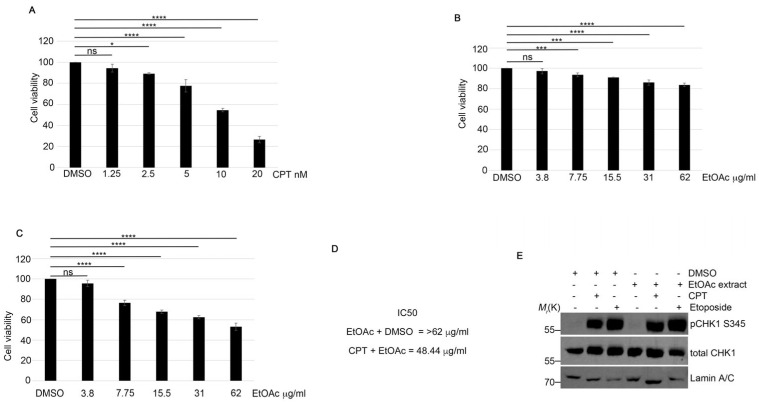
Fermented grape extracts increased the efficacy of CPT treatment. (**A**) HeLa cells were treated with CPT at the indicated concentrations and incubated for 72 h. Cell viability was assessed using a standard assay. Statistical analysis was performed using a one-way repeated measures ANOVA with a multiple comparison post-test to compare all groups. Statistically significant differences are indicated as * *p* < 0.05 and **** *p* < 0.0001. (**B**) HeLa cells were treated with EtOAc extract in combination with DMSO and incubated as described in (**A**). *** *p* < 0.001 and **** *p* < 0.0001. (**C**) HeLa cells were treated with 2.5 nM CPT and various concentrations of EtOAc extract for 72 h. Cell viability was measured using assay. **** *p* < 0.0001. (**D**) IC50 values for EtOAc + DMSO and EtOAc + CPT were calculated using Prism 9 software. (**E**) HeLa cells were pre-treated with 0.63 μg/mL of EtOAc extract for one hour, followed by incubation with 1 μM CPT or 20 μM Etoposide for an additional two hours. Cells were then lysed, and Western blot analysis was performed using the indicated antibodies. Lamin A/C was used as a protein loading control.

To identify potential new molecules for cancer treatment, we analyzed the effects of lyophilized fermented Aglianico red grape pomace extracted with ethyl acetate (EtOAc) on HeLa cell viability. First of all, we used a CPT treatment to induce DSBs and HR repair (Figure 1A), and we observed a reduction of approximately 10% in cell viability at a CPT concentration of 2.5 nM (Figure 1A). To rule out any potential effects of DMSO, the solvent used to dissolve the EtOAc grape pomace extract and CPT, we conducted additional treatments with DMSO in combination with previous compounds (Figure 1A,B). As shown in Figure 1C, the combination of the EtOAc grape pomace extract at various concentrations with 2.5 nM CPT reduced the cell viability in all treatments compared to CPT alone. We focused on analyzing the impact of the grape extract on DDR modulation following CPT treatment. Firstly, we monitored the activation of Checkpoint Kinase 1 (CHK1) via its phosphorylation at Serine 345 (pCHK1 S345) [11], which is activated upon CPT treatment. As shown in Figure 1D, CPT induced CHK1 S345 activation similarly to the EtOAc combination. Additionally, we evaluated the effects of another DNA double-strand break agent, etoposide, a topoisomerase II inhibitor [12], in combination with the EtOAc extract, again finding no differences in pCHK1 activation (Figure 1D and Figure S1A).

**Figure 2:**
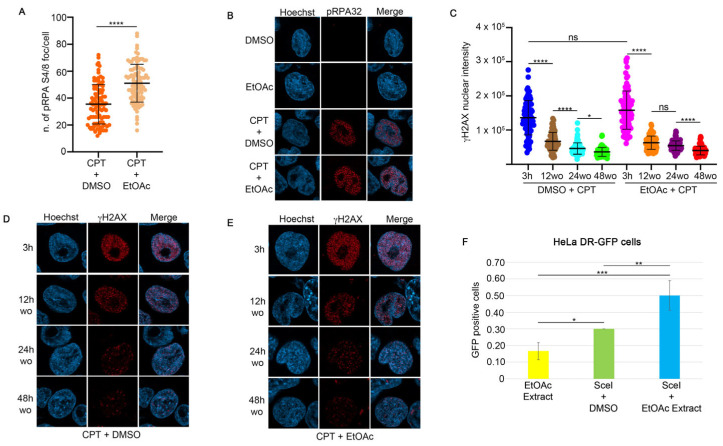
EtOAc does not affect DNA damage repair ability induced by camptothecin. (**A**) RPA32 S4/8 foci intensity in HeLa cells treated for one hour with EtOAc at 0.63 μg/mL, followed by incubation with 1 μM CPT for an additional two hours. Foci number was measured with Fiji software. **** *p* < 0.0001. (**B**) Representative images of pRPA32 following incubation with the indicated reagents. All immunofluorescence images were acquired with the LSM900 using AiryScan2. Magnification 63×. (**C**) γH2AX intensity was measured in HeLa cells pre-treated with EtOAc extract or DMSO for one hour, followed by 1 μM CPT treatment. At the end of incubation time we performed a drugs washout, monitoring the DNA repair as a measure of γH2AX nuclear signal. Immunofluorescence images were analyzed with Fiji software. * *p* < 0.05 and **** *p* < 0.0001. (**D**) Representative images of H2AX S139 nuclear intensity in HeLa cells treated with 1 μM CPT and DMSO for three hours, followed by drug washout to monitor H2AX recovery. Magnification 63×. (**E**) Immunofluorescence analysis of HeLa cells treated for one hour with EtOAc at 0.63 μg/mL, followed by incubation with 1 μM CPT for an additional two hours. Magnification 63×. (**F**) HeLa cells stably transfected with the pDR-GFP vector were co-transfected with I-SceI endonuclease and incubated with 0.63 μg/mL of EtOAc extract for 24 h. HR activity was measured using FACS analysis, with GFP levels serving as an indicator of HR frequency. Data are presented as means ± standard deviation from three independent experiments. Statistically significant differences are indicated by * *p* < 0.05, ** *p* < 0.01, and *** *p* < 0.001.

**Figure 3:**
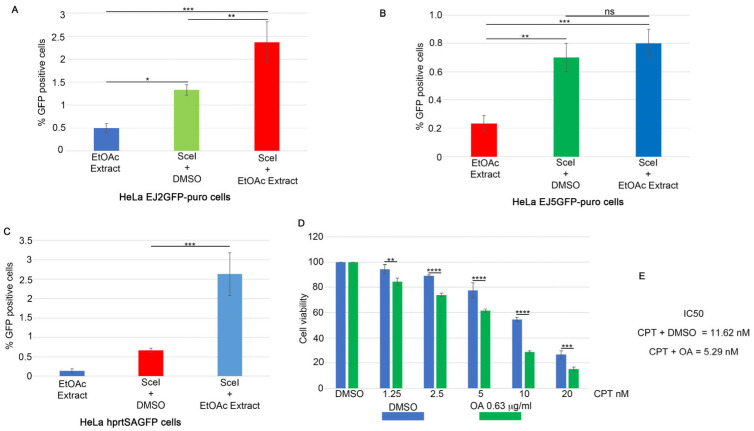
OA reduces HeLa cell viability in combination with CPT. (**A**) HeLa cells stably expressing the EJ2GFP-puro reporter plasmid were transfected with the SceI plasmid, followed by incubation with EtOAc for 24 h. At the end of the incubation period, GFP levels were measured using FACS analysis to assess Alt-EJ frequency. Data are shown as means ± standard deviation from three independent experiments. Statistically significant differences are indicated by * *p* < 0.05, ** *p* < 0.01 and *** *p* < 0.001. (**B**) HeLa cells stably expressing the pimEJ5GFP reporter plasmid were transfected with the I-SceI coding plasmid, followed by incubation as described in (**A**). NHEJ activity was measured using FACS analysis, with GFP levels serving as an indicator of NHEJ frequency. Data are presented as means ± standard deviation from three independent experiments. Statistically significant differences are indicated by ** *p* < 0.01, *** *p* < 0.001. (**C**) HeLa cells carrying the SSA-GFP reporter plasmid were transfected with the I-SceI endonuclease and treated as described in (**A**). Statistically significant differences are indicated by *** *p* < 0.001. (**D**) OA at 0.63 μg/mL or DMSO was pre-incubated for one hour, followed by incubation with different concentrations of CPT for 72 h. The mean of three independent experiments is reported, followed by statistical analysis. ** *p* < 0.01, *** *p* < 0.001, **** *p* < 0.0001. (**E**) IC50 calculation, using GraphPad software, from cell viability assay of HeLa cells treated as described in (**D**).

To monitor the effect of CPT on the activation of HR, we assessed the foci intensity of RPA32 S4/8 phosphorylation (pRPA32 S4/8), a marker of DNA end-resection [13], in HeLa cells treated with CPT alone or in combination with the EtOAc extract. As shown in Figure 2A,B, the CPT/EtOAc co-treatment increased pRPA32 S4/8 levels compared to CPT alone. Moreover, to test if the presence of EtOAc affects the activation of H2AX at Serine 139, we analyzed its nuclear intensity in response to CPT or in combination with EtOAc, showing no differences after 3 h of treatment (Figure 2C) [14]. To analyze the possible effect of EtOAc on DNA repair kinetics during CPT treatment, we pre-treated HeLa cells with EtOAc or DMSO for one hour, followed by incubation with CPT for an additional two hours (Figure 2C–E and Figure S1B,C). At the end of the incubation, we measured the H2AX nuclear intensity as a maximum marker of DNA damage induced by CPT (3 h). At this time, drug treatments were washed out, followed by incubation with a complete cell medium to monitor DNA repair efficiency. As reported in Figure 2C, EtOAc does not affect the repair efficiency after CPT treatment. To investigate which DNA repair mechanisms were activated in response to the CPT/EtOAc co-treatment, we used HeLa cells stably expressing reporter plasmids to measure the frequencies of GC (pDR-GFP) [15], SSA (hprtSAGFP) [16], Alt-EJ (EJ2GFP-puro) [17], and c-NHEJ (Transfection of SceI endonuclease (pCBA-SceI)) [18] into HeLa cells with a non-functional GFP reporter plasmid inducing DSBs in the plasmid sequence, leading to correct DNA repair and GFP emission, which was measured using an FACS analysis. Treatment with the EtOAc extract resulted in a slight increase in HR frequencies (Figure 2F) and Alt-EJ frequencies (Figure 3A), but not an increase in NHEJ activity (Figure 3B) [17]. Surprisingly, this extract upregulated the SSA activity four-fold compared to the vehicle control (Figure 3C).

### 2.2. EtOAc Extract Analysis Through Nuclear Magnetic Resonance Spectroscopy (NMR) Reveals the Presence of Oleanolic Acid and 9(Z),11(E)-Conjugated Linoleic Acid Ethyl Ester

An NMR-based analysis was conducted to identify the major bioactive molecules in the extract obtained from Aglianico red grape pomace using EtOAc as a solvent. The ^1^H NMR spectrum of the extract revealed several metabolites, with OA being the most abundant, apart from glycerol (Figure 2E). The unambiguous identification of this triterpenoid was achieved by comparing its NMR chemical shifts with those reported in the literature [19]. The quantification of OA was performed using NMR with pyridine as an internal standard, chosen because its proton resonances do not overlap with any signals from the metabolites in the analyzed fraction. The amount of OA in the extract was determined to be 0.33 ± 0.04 mg. The NMR analysis, alongside OA, also identified additional metabolites including glycerol, malic acid, syringic acid, and gallic acid. These compounds were identified by comparing their resonances with values in the literature [20], but they were present in quantities too small for accurate quantification. Additionally, NMR signals in the olefinic proton region led to the identification of a spin system corresponding to 9(Z),11(E)-conjugated linoleic acid (CLA) (Figure S1D). LC-MS/MS analysis confirmed that the identified molecule was the ethyl ester of CLA. Structural elucidation was based on the fragmentation pattern of the ion peak at m/z 309.3, with major fragment ions detected at m/z 263.3 and m/z 245.3. This MS pattern matched that reported by Gong et al. [20]. The presence of CLA as an ethyl ester is likely a product of yeast metabolism during winemaking, which may have led to the esterification of carboxylic moieties with ethanol. The CLA ethyl ester was quantified using NMR at 0.06 ± 0.04 mg, using the same method as for OA.

### 2.3. Oleanolic Acid Increases the Activity of CPT in the HeLa Cell Line

To investigate the effects of OA and CLA on HeLa cells, we performed a cell viability assay. As shown in Figure 1C, 2.5 nM of CPT increased its activity in the presence of 7.7 µg/mL of the total grape pomace extract. In Figure S1A, we showed that the amounts of OA and CLA in the extract were 0.33 mg and 0.06 mg, respectively, per 1 mg of EtOAc extract (dry weight). We tested OA concentrations ranging from 0.63 to 10 µg/mL, with 2.5 µg/mL being the concentration in the 7.7 µg/mL dose of the total EtOAc extract. We observed a reduction in cell viability starting at an OA concentration of 5 µg/mL (Figure S1D). Consequently, we combined OA at 0.63 µg/mL, which does not affect cell proliferation, or DMSO with varying concentrations of CPT and incubated for 72 h. Figure 3D shows that the presence of OA enhanced the efficacy of CPT compared to the vehicles. Additionally, we treated HeLa cells with CLA at concentrations ranging from 0.11 to 1.8 µg/mL, with 0.46 µg/mL corresponding to the amount in the 7.7 µg/mL pomace extract. This treatment showed no consistent effect on cell viability across all concentrations (Figure S2A). Combining CLA at 0.11 µg/mL with CPT showed no significant differences compared to DMSO (Figure S2B). These data suggest that OA can enhance the efficacy of CPT in HeLa cell lines.

### 2.4. OA Drives the Resolution of DSBs Through Single-Strand Annealing

**Figure 4:**
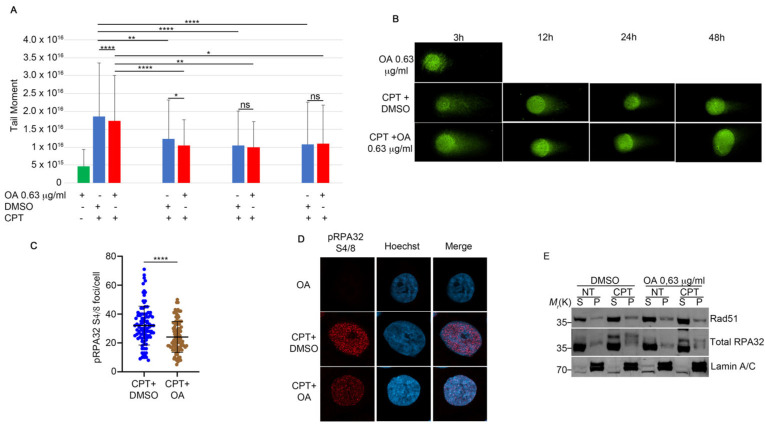
OA treatment does not affect the DNA repair ability compared to CPT alone. (**A**) The neutral comet assay was performed as described in Section 4. HeLa cells were pre-treated with OA or DMSO, followed by incubation with 1 μM CPT for an additional two hours. At the end of the incubation period, a drug washout was performed to monitor DNA repair. For each condition, 5 × 10⁴ cells were spotted onto glass slides and stained with SYBR Green. We analyzed 30 cells for each condition. The results show the means and standard deviation (SD) of three independent experiments. Statistically significant differences are indicated by * *p* < 0.05, ** *p* < 0.01, and **** *p* < 0.0001. (**B**) Representative images of the neutral comet assay. Magnification 63×. (**C**) Immunofluorescence analysis of pRPA32 S4/8 foci per cell in HeLa cells treated with CPT alone or in combination with OA. Immunofluorescence staining was performed with pRPA32 S4/8, and Hoechst was used as a DNA marker. We analyzed 30 cells for each condition across three independent experiments. Statistical analysis was performed using a *t*-test, and significant differences are indicated by **** *p* < 0.0001. (**D**) Representative images of pRPA32 S4/8 in HeLa cells treated as described in (**C**). Magnification 63×. (**E**) HeLa cells were pre-treated with 0.63 µg/mL of OA for one hour, followed by incubation with 1 µM CPT for an additional two hours. Chromatin-enriched purification was performed to separate soluble (S) and chromatin-bound (P) fractions. RPA32 protein was used as a marker for the soluble fraction and as a control for DNA damage, while Lamin A/C served as a loading control for the chromatin fraction.

**Figure 5:**
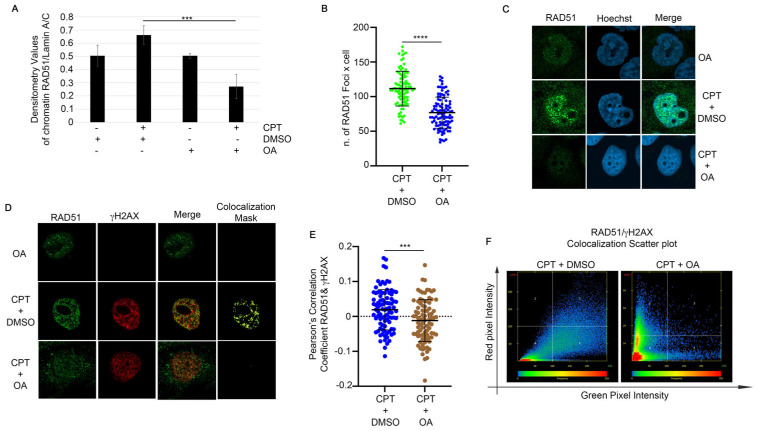
Oleanolic acid reduces RAD51 localization onto DNA damage sites. (**A**) Densitometric analysis of RAD51 chromatin loading from three independent experiments. The analysis was performed using Fiji software by dividing the densitometry values for RAD51 by the loading control, Lamin A/C. *** *p* < 0.001. (**B**) RAD51 foci were analyzed in HeLa cells pre-treated with OA and CPT as described in (**A**), using Fiji software. The results are shown as means ± standard deviation (SD) from three independent experiments, with 30 cells analyzed per condition. Statistical differences were assessed using the Student’s *t*-test and are indicated as **** *p* < 0.0001. (**C**) Representative images of RAD51 foci formation in HeLa cells treated as described in (**B**). Magnification 63×. (**D**) RAD51 and γH2AX colocalization was assessed in HeLa cells treated as described in (**B**). Images were acquired using Zen software, and a colocalization mask was generated to visualize the overlap between RAD51 and γH2AX foci. Magnification 63×. (**E**) Pearson’s correlation coefficient was calculated using Zen software to quantify the colocalization of RAD51 and γH2AX in HeLa cells pre-treated as in (**A**). The results are presented as means ± SD from three independent experiments, with 30 cells analyzed per condition. Statistical differences were evaluated using the Student’s *t*-test and are indicated as *** *p* < 0.001. (**F**) A scatter plot depicting RAD51 and γH2AX colocalization in HeLa cells treated as described in A. Images were obtained using Zen software, showing the distribution and overlap of RAD51 and γH2AX foci.

**Figure 6:**
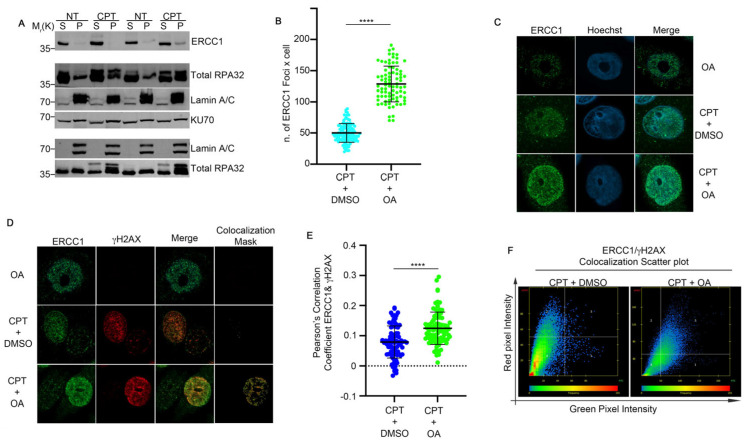
ERCC1 localized to DNA damaged sites upon OA treatment. (**A**) HeLa cells were pre-treated with Oleanolic Acid (OA) at 0.63 µg/mL or DMSO for one hour, followed by incubation with 1 µM CPT for two hours. Chromatin purification was performed to separate soluble (S) and chromatin-bound (P) fractions. Total RPA32 was used for normalization of the soluble fraction (S) and as a DNA damage control, while Lamin A/C served as a loading control for the chromatin fraction. (**B**) Immunofluorescence analysis of HeLa cells treated as described in (**A**) was conducted to assess ERCC1 foci formation. Foci were quantified using Fiji software. The graph shows means ± standard deviation (SD) from three independent experiments, with 30 cells analyzed per condition. Statistical significance was determined by Student’s *t*-test, with results indicated as **** *p* < 0.0001. (**C**) Representative images of ERCC1 foci from HeLa cells treated as described in (**A**) are shown. Images were acquired using Zen software. Hoechst staining was used as a DNA marker to visualize cell nuclei. Magnification 63×. (**D**) Immunofluorescence images showing the colocalization of ERCC1 and γH2AX in HeLa cells treated as in (**A**). Colocalization masks were generated using Zen software to illustrate the overlap between ERCC1 and γH2AX foci. (**E**) Pearson’s correlation coefficient was calculated using Zen software to quantify the colocalization of ERCC1 and γH2AX in HeLa cells treated as described in (**A**). The graph shows means ± SD from three independent experiments, with 30 cells analyzed per condition. **** *p* < 0.0001. (**F**) A scatter plot depicting ERCC1 and γH2AX colocalization in HeLa cells treated with OA or DMSO for one hour, followed by CPT treatment, is presented. Images were analyzed with Zen software to visualize the distribution and overlap of ERCC1 and γH2AX foci.

We reported in Figure 2 that the EtOAc extract induced the modulation of pRPA32 protein and H2Ax. First of all, we checked if OA, in combination with CPT, was able to induce an alteration of DSBs through the analysis of a comet assay. As reported in Figure 4A,B, OA did not modify the comet tail moment, which is the tail length × % of DNA in the tail [21], in combination with CPT when compared with the topoisomerase inhibitor alone (Figure 4A,B). Moreover, the analysis of pRPA32 revealed a reduction in the CPT-induced foci intensity in the presence of OA when compared with the control (Figure 4C,D). To investigate the molecular biology processes regulated by OA in CPT-treated cells, we performed a chromatin fraction assay to examine RAD51 loading onto chromatin, a key regulator of GC [22]. HeLa cells were pre-treated with 0.63 µg/mL of OA or DMSO for one hour, followed by 1 µM CPT for an additional two hours. As shown in Figure 4E and Figure 5A, CPT increased the RAD51 chromatin binding, but the pre-treatment with OA reduced its loading. RAD51, a biomarker of HR, and its foci formation serve as an indicator of HR activity [23]. An immunofluorescence (IF) analysis of RAD51 foci in HeLa cells treated with CPT alone or in combination with OA revealed a reduction in the number of its foci with OA pre-treatment compared to CPT alone (Figure 5B,C). Additionally, we evaluated the colocalization of RAD51 with γH2AX using Pearson’s correlation coefficient. The correlation coefficients were 0.019 for CPT + DMSO and −0.011 for CPT + OA, as determined using confocal IF (Figure 5D–F) [24]. pRPA32 was reduced upon OA treatment (Figure 4C), suggesting a potential resolution of DNA damage through another repair pathway. As reported in the literature, RPA32 is crucial for gene conversion and SSA [25]; we investigated whether OA drives DNA repair through SSA. First of all, we analyzed ERCC1 chromatin loading, a regulator of SSA [26], in HeLa cells treated with CPT alone or combined with OA. Figure 6A and Figure S2C show that the OA pre-treatment induced a marked increase in ERCC1 chromatin loading compared to CPT alone, but not for KU70, an HNEJ protein regulator (Figure 6A and Figure S2D) [27]. Consistent with this, the immunofluorescence of ERCC1 foci increased in OA pre-treated cells (Figure 6B,C). Furthermore, the Pearson’s correlation coefficient of ERCC1 and γH2AX colocalization increased with the OA pre-treatment (Figure 6D–F). These data support the hypothesis that OA drives the HR repair induced by CPT through single-strand annealing.

## 3. Discussion

Natural extracts from various vegetal substrates represent a promising source of molecules that could be instrumental in developing new cancer treatments. These compounds offer significant potential for enhancing the efficacy of personalized targeted therapies and may provide novel strategies to counteract emerging drug resistance mechanisms [28]. In this study, we examined the effects of a fermented extract from Aglianico grape pomace, a by-product of industrial wine production, extracted with EtOAc solvent, on the HeLa cancer cell line, both alone and in combination with CPT. Cell viability assays in HeLa cells showed an increased sensitivity to CPT, indicating a potential modulation of the DNA damage response. Interestingly, our investigation into the DNA repair pathways affected by the EtOAc extract revealed a significant increase in SSA activity, along with a slight increase in HR and Alt-EJ. These effects may be attributed to the diverse range of molecules present in the total extract, each potentially targeting different aspects of the DNA repair mechanisms. To identify specific active components within the EtOAc extract, we conducted an NMR-based analysis, which identified and quantified two major metabolites: OA and, at a much lower concentration, CLA. Notably, CLA has been previously associated with increased anti-inflammatory gene expression in mouse cell lines [23]. Conversely, OA has been shown to reduce cancer cell proliferation and tumor growth in mice when used in combination with radiotherapy and Olaparib [10]. In our cell viability assays, OA induced cell death starting from 5 µg/mL after 72 h of incubation, while CLA did not affect cell viability at any concentration tested. The EtOAc grape pomace extract at 7.7 µg/mL reduced HeLa cell viability by approximately 20%. According to our NMR analysis, this concentration included 2.5 µg/mL of OA, which alone did not affect cell viability. This suggests that the observed effects might be due to the synergistic action of various compounds in the EtOAc extract.

Our goal was to identify a molecule from natural extracts that would not be cytotoxic when used alone but could enhance the efficacy of cancer therapies when combined with them. We tested 0.11 µg/mL of CLA and 0.63 µg/mL of OA—concentrations that do not affect cell survival—combined with various doses of CPT. Only the combination of OA with CPT resulted in an increased sensitivity compared to CPT alone. Moreover, the analysis of the comet assay revealed no difference in genome stability when CPT was combined with OA, suggesting that the increased cell sensitivity to OA in the presence of the DNA-damaging agent was due to an alteration in the DNA repair mechanisms.

Based on these findings, we further investigated OA’s role in cancer therapy. CPT induces DNA damage in replicating cells, primarily repaired by HR, which is regulated by RAD51 [23]. Our analysis showed reduced RAD51 chromatin binding in the presence of OA. Immunofluorescence confocal analysis revealed a decreased colocalization of RAD51 with γH2AX foci in OA-treated cells. In contrast, OA promoted repair via the SSA pathway, as indicated by the increased ERCC1 loading on chromatin. Consistently, we analyzed the chromatin loading of KU70 in HeLa damaged cells, in the presence of OA, and found a reduction in protein chromatin loading, suggesting a decreased activation of c-NHEJ, in which ERCC1 favors the end-ligation process [29]. Moreover, the analysis of pRPA S4/8 in HeLa cells treated with CPT and OA revealed a reduced foci intensity compared to CPT alone. This suggests that DNA repair may occur through a faster process, such as SSA, rather than homologous recombination (HR), which requires DNA synthesis for proper repair. Overall, our results revealed that OA can effectively modulate DNA repair pathways in HeLa cells, promoting a more mutagenic repair process and enhancing the efficacy of cancer therapy. As reported in several studies, SSA is an important repair pathway characterized by large DNA deletions, making it a more mutagenic repair mechanism, which can increase cell death [1,2,3]. In line with this evidence, CPT treatment and DMSO favor HR repair, whereas cotreatment with OA promotes the more mutagenic SSA, thereby enhancing CPT-induced cytotoxicity. The non-cytotoxic nature of OA at low doses, combined with its ability to increase CPT efficacy, may reduce the side effects associated with CPT treatment. This supports the previously described role of OA [10] and highlights its potential as a novel combinational agent in cancer therapy.

## 4. Materials and Methods

### 4.1. Cell Culture, DNA Constructs, and Transfection

The HeLa cell line was obtained from the American Type Culture Collection (ATCC, CCL-2) and cultured in Roswell Park Memorial Institute (RPMI) 1640 medium (Thermo Fisher Scientific, Waltham, MA, USA). The medium was supplemented with 10% fetal bovine serum (FBS, Thermo Fisher Scientific), penicillin (100 U/mL), streptomycin (100 µg/mL), and 2 mM glutamine. Cells were maintained at 37 °C in a humidified atmosphere containing 5% CO₂.

### 4.2. Antibodies and Western Blotting

The following antibodies were used: RPA32 (1:5000, A300–244A, Bethyl Laboratories, Montgomery, TX, USA), RPA32 S4/S8 (1:2000, A300–245A, Bethyl Laboratories), Lamin A/C (1:1000, #4777, Cell Signalling, Danvers, MA, USA), CHK1 S345 (1:1000, #2348, Cell Signalling), CHK1 (1:1000, #2360, Cell Signalling), GAPDH (1:1000, sc-25778, Santa Cruz Biotechnology, Dallas, TX, USA), RAD51 (1:1000, NB100-148, Novus Biologicals, Centennial, CO, USA), and KU70 (1:500, sc-1486, Santa Cruz Biotechnology). For total protein extraction, cells were lysed on ice at 4 °C in a buffer containing 50 mM HEPES (pH 7.5), 1% Triton X-100, 150 mM NaCl, and 5 mM EGTA, supplemented with a protease and phosphatase inhibitor cocktail (Roche Applied Science, Penzberg, Germany). The lysates were clarified using centrifugation at 10,000× *g* for 20 min at 4 °C and subjected to SDS-PAGE. Chemiluminescent images were captured using the ImageQuant LAS 500 (GE Healthcare, Chicago, IL, USA). Densitometric analysis of phosphorylated proteins was performed as follows: total protein levels were first normalized to the loading control, and then phosphorylated protein levels were normalized to the corresponding total protein.

### 4.3. Cell Fractionation

Cell fractionation was performed with minor modifications to previously described methods [30]. Briefly, 3 × 10^6^ cells per condition were collected and resuspended in 200 µL of CSK buffer (10 mM PIPES, pH 6.8, 100 mM NaCl, 300 mM MgCl₂, 1 mM EGTA, 1 mM DTT, 0.1% Triton X-100, 0.34 M sucrose) supplemented with protease and phosphatase inhibitors. The cell suspension was incubated on ice for 5 min. The soluble cytoplasmic fraction (S) was separated from the nuclei (P) using centrifugation at 1300× *g* for 4 min at 4 °C. The nuclear pellet (P) was washed with CSK buffer, then resuspended in 200 µL of Western blot buffer. This suspension was sonicated and centrifuged for 30 min at 10,000× *g* at 4 °C. The supernatant from the centrifugation was collected, and both fractions (cytoplasmic and nuclear) were subjected to SDS-PAGE and analyzed using Western blot using the indicated antibodies.

### 4.4. Microscope Image Acquisition

HeLa cells were grown on glass coverslips and fixed with 4% paraformaldehyde. Following fixation, cells were permeabilized with 0.2% Triton X-100. Blocking was performed for 10 min at room temperature (RT) using 1% BSA. Cells were then incubated for 1 h at 37 °C with primary antibodies: anti-RAD51 (1:200, sc-8349, Santa Cruz Biotechnology) anti-RPA32 S4/8 (1:200, A300-245A, Bethyl Laboratories), anti-ERCC1 (1:200, NB500-704, Novus Biological, Centennial, CO, USA), γH2AX S-139 (1:200, ab2893, Abcam, Cambridge, UK), and γH2AX S-139 (1:200, 05-636, Millipore, Burlington, MA, USA). After washing, cells were incubated for 45 min at 37 °C with AlexaFluor 647 donkey ant-mouse IgG (H+L) (ThermoFisher Scientific), AlexaFluor 647 donkey ant-rabbit IgG (H+L) (ThermoFisher Scientific), AlexaFluor 488 goat anti-mouse IgG (H+L) (ThermoFisher Scientific), and AlexaFluor 488 goat anti-rabbit IgG (H+L) (ThermoFisher Scientific), and mounted with ProLong™ Gold Antifade Mountant with DNA Stain DAPI (Thermofisher Scientific) onto glass slides. Microscope image acquisition was conducted using a Zeiss LSM 900 AiryScan2 with a 63×/1.4 oil objective (Zeiss, Oberkochen, Germany) and images were captured with Zen 3.9 software (Zeiss, Oberkochen, Germany). Foci intensity per cell was quantified using Fiji software (version 2.14.0/1.54f).

### 4.5. Cell Viability Assay

HeLa cells were seeded in triplicates in 96-well plates at a density of 1500 cells/well and allowed to adhere for 24 h. Cells were treated at the indicated drug concentrations and incubated for an additional 72 h. At the end of the treatment, cells were fixed with 50% *v*/*v* trichloroacetic acid and stained with 0.4% *w*/*v* sulforhodamine B (SRB) in 1% *v*/*v* acetic acid. The percentage of cell viability after treatment was calculated assuming 100% of the number of untreated cells. For cell viability assay, data represent means ± standard deviation (n = 3 independent experiments). Data were subjected to multiple one-way repeated measures ANOVA tests with multiple comparison post-test to compare all groups.

### 4.6. DNA Reporter Assays

HeLa-pDRGFP cells [31], stably expressing the DR-GFP reporter plasmid, were cotransfected with a plasmid encoding the I-SceI endonuclease and the indicated siRNAs or vectors. After 48 h of incubation, GFP expression, indicative of homologous recombination (HR) frequency, was analyzed using FACS. Similarly, HeLa cells stably expressing the pimEJ5GFP reporter plasmid were cotransfected with the I-SceI endonuclease plasmid and the indicated siRNAs or vectors. After 48 h of incubation, GFP expression, reflecting non-homologous end joining (NHEJ) frequency, was assessed using FACS analysis. pDRGFP was a gift from Maria Jasin (Addgene plasmid #26475). hprtSAGFP was a gift from Maria Jasin (Addgene plasmid #41594). EJ2GFP-puro was a gift from Jeremy Stark (Addgene plasmid #44025). pCBASceI was a gift from Maria Jasin (Addgene plasmid # 26477). pimEJ5GFP was a gift from Jeremy Stark (Addgene plasmid #44026).

### 4.7. Chemicals and Reagents

All solvents used in this study were of HPLC grade or higher and were purchased from Sigma-Aldrich (Milan, Italy). Aqueous solutions were prepared with Milli-Q water from Millipore (Bedford, MA, USA). Deuterated solvents for NMR-based analyses were purchased from Cambridge Isotope Laboratories, Inc. (Tewksbury, MA, USA).

### 4.8. Grape Pomace Collection, Extraction, and Chromatographic Separation

Aglianico (*Vitis vinifera* L. cv) grape pomace was obtained after winemaking procedures in November of 2021. Grapes were from the Taurasi area in Avellino, Italy. Then, 1.0 g samples of lyophilized pomace were separately extracted overnight with 50 mL of five different solvents including Acetone, Butanol (BuOH), Ethyl Acetate (EtOAc), Dimethyl Carbonate (DMC), and 2-methyltetrahydrofuran (2-MeTHF). The extracts were evaporated under vacuum.

### 4.9. NMR and MS Analyses

^1^H (600 MHz) NMR spectra were measured on a Bruker spectrometer by using a Norell Select Series 5 mm NMR tube (Norell, Inc., Morganton, NC, USA). Chemical shifts were referenced to the residual solvent signal (CD_3_OD: δ_H_ 3.31, δ_C_ 49.3 ppm). NMR data of OA are reported in Errichiello et al. [32]. For quantitation purposes, 5 μL of pyridine anhydrous (99.8%; Sigma-Aldrich, Milan, Italy) was used as an internal standard. ^1^H-NMR spectra were then acquired with a d1 value of 7.0 s. Measurements were performed in triplicate. To quantify OA, NMR signals attributed to the methyl groups were selected. To quantify CLA ethyl ester, resonances of its olefinic protons were chosen. The selected NMR signals were measured using integration and divided by the number of protons. Conversion into the relative number of moles was conducted by comparing the measured areas of the selected NMR resonances with those generated by the pyridine protons, for which the number of moles was known. LC-MS/MS analyses were performed on an Agilent Ultivo Triple Quadrupole coupled with an HPLC Agilent 1260 infinity II LC apparatus (Santa Clara, CA, USA) consisting of a binary pump and two-channel degasser unit. Chromatographic separation was obtained on a Kinetex 2.6 um C18 (100 × 2.1) column (Phenomenex, Torrance, CA, USA) eluted with a 20 min linear gradient from 40 to 100% of B (Buffer A: H_2_O 0.01% Formic Acid and Buffer B: Acetonitrile 0.01% Formic Acid). The ESI source was operated in both positive and negative ion modes and the analyzer was operated in Selected Reaction Monitoring (SRM) mode. Source conditions were as follows: spray voltage: 3.5 kV (positive mode) and 2.9 kV (negative mode); capillary voltage: 25 V; source temperature: 320 °C; normalized collision energy: 30 AU. The acquisition range was *m*/*z* 100–1000. For the identification of OA, the SRM transition was 455.3 → 407.3 in negative ion mode (Rt = 14.85 min); for CLA ethyl ester, the SRM transition was 309.3 → 263.3 in positive ion mode (Rt = 15.90 min).

### 4.10. Neutral Comet Assay

Neutral comet assay was performed as described previously [33]. Briefly, HeLa cells were pre-treated with oleanolic acid (OA) at 0.63 µg/mL or DMSO for one hour, followed by incubation with camptothecin (CPT) at 1 µM for an additional two hours. At the end of the incubation period, 5 × 10⁴ cells were resuspended in 0.6% low melting agarose (Sigma Aldrich, St. Louis, MO, USA) and spotted onto glass slides, followed by incubation for one hour at 4 °C with lysis solution (2.5 M NaCl, 0.1 M EDTA, 10 mM Tris-HCl pH 10, 0.5% Triton X-100). Subsequently, the glass slides were washed three times with electrophoresis running buffer (Tris/Acetic Acid/EDTA buffer). Finally, the slides were placed in an electrophoresis chamber and run at 0.6 V/cm for 30 min at room temperature, followed by washing with PBS 1X. Cell comets were incubated with SYBR Green (ThermoFisher Scientific) for 15 min at room temperature, then washed twice with PBS 1X and once with 70% ethanol, followed by air drying overnight at room temperature. Comet analysis was performed using Open Comet software (version v1.3.1) [34].

### 4.11. Statistical Analysis

Statistical analysis was performed using the GraphPad Prism Software, version 9 for Mac. To evaluate differences between the means of two groups, we used a two-sided Student *t*-test, whereas to analyze differences among the means of multiple groups we used one-way ANOVA with Kruskal–Wallis post-test. *p* < 0.05 was considered to be statistically significant. The number of independent experiments and *p*-values are reported in the figure legends.

## Data Availability

The data presented in this study are openly available in [Zenodo] at [https://zenodo.org/], reference number [12545580].

## Supplementary Materials

The following supporting information can be downloaded at: https://www.mdpi.com/article/10.3390/ijms252413475/s1.
