# Supplementary material for: Oleanolic Acid Modulates DNA Damage Response to Camptothecin Increasing Cancer Cell Death
Source: Int J Mol Sci. 2024 Dec 16;25(24):13475. doi: 10.3390/ijms252413475 (PMC11676975; doi:10.3390/ijms252413475)
Supplement: Supplementary file 1 [file ijms-25-13475-s001.zip › ijms-3272451-supplementary legend.pdf]

Supplementary Figure Legend

Supplementary Figure S1. A) Densitometric analysis of CHK1 S345 phosphorylation in response to EtOAc treatment. HeLa cells were pretreated with EtOAc for one hour, followed by incubation with CPT or Etoposide for an additional two hours. Densitometry was performed using Fiji software, where the values of total CHK1 were divided by the protein loading control, Lamin A/C. The pCHK1 value was then normalized to the normalized total CHK1. Data are presented as means ± standard deviation from three independent experiments. Statistically significant differences are indicated by $*P < 0.05$. **B)** Representative images from the immunofluorescence assay of HeLa cells treated with DMSO for three hours, followed by washout and incubation at the indicated time points. Magnification 63x. **C)** Immunofluorescence assay of HeLa cells treated with EtOAc as in panel B. Magnification 63x. **D)** The ^1H NMR spectrum of the EtOAc extract was analyzed to identify its major metabolites. Oleanolic acid was identified as the most abundant metabolite. The enlargement of the spectrum shows the resonances of olefinic protons, which were assigned to CLA. A table summarizing the most abundant compounds identified in the EtOAc extract is provided. **E)** HeLa cells were treated with varying concentrations of OA for 72 hours. Cell viability was assessed as described in the Materials and Methods section. The results are presented as the means and standard deviation (SD) of three independent experiments. Cell viability is expressed as a percentage relative to control cells treated with DMSO alone. Statistically significant differences are indicated by $*P < 0.05$.

Supplementary Figure S2. A) HeLa cells were treated with different concentrations of CLA, followed by a cell viability assay after 72 hours of incubation. Statistically significant differences are indicated by *$P < 0.05$. **B)** HeLa cells were treated with 0.11 μg/ml of CLA or vehicle for one hour, followed by CPT treatment at different concentrations. Cell viability was assessed as described in the Materials and Methods section. Statistically significant differences are indicated by *$P < 0.05$. **C)** Densitometric analysis of ERCC1 chromatin loading from three independent experiments. Densitometry was performed using Fiji software, normalizing the ERCC1 protein values to the Lamin A/C values. **D)** Densitometric analysis of KU70 chromatin loading, analyzed as described in panel C.
