# Supplementary figures and images for: Oleanolic Acid Modulates DNA Damage Response to Camptothecin Increasing Cancer Cell Death
Source: Int J Mol Sci. 2024 Dec 16;25(24):13475. doi: 10.3390/ijms252413475 (PMC11676975; doi:10.3390/ijms252413475)

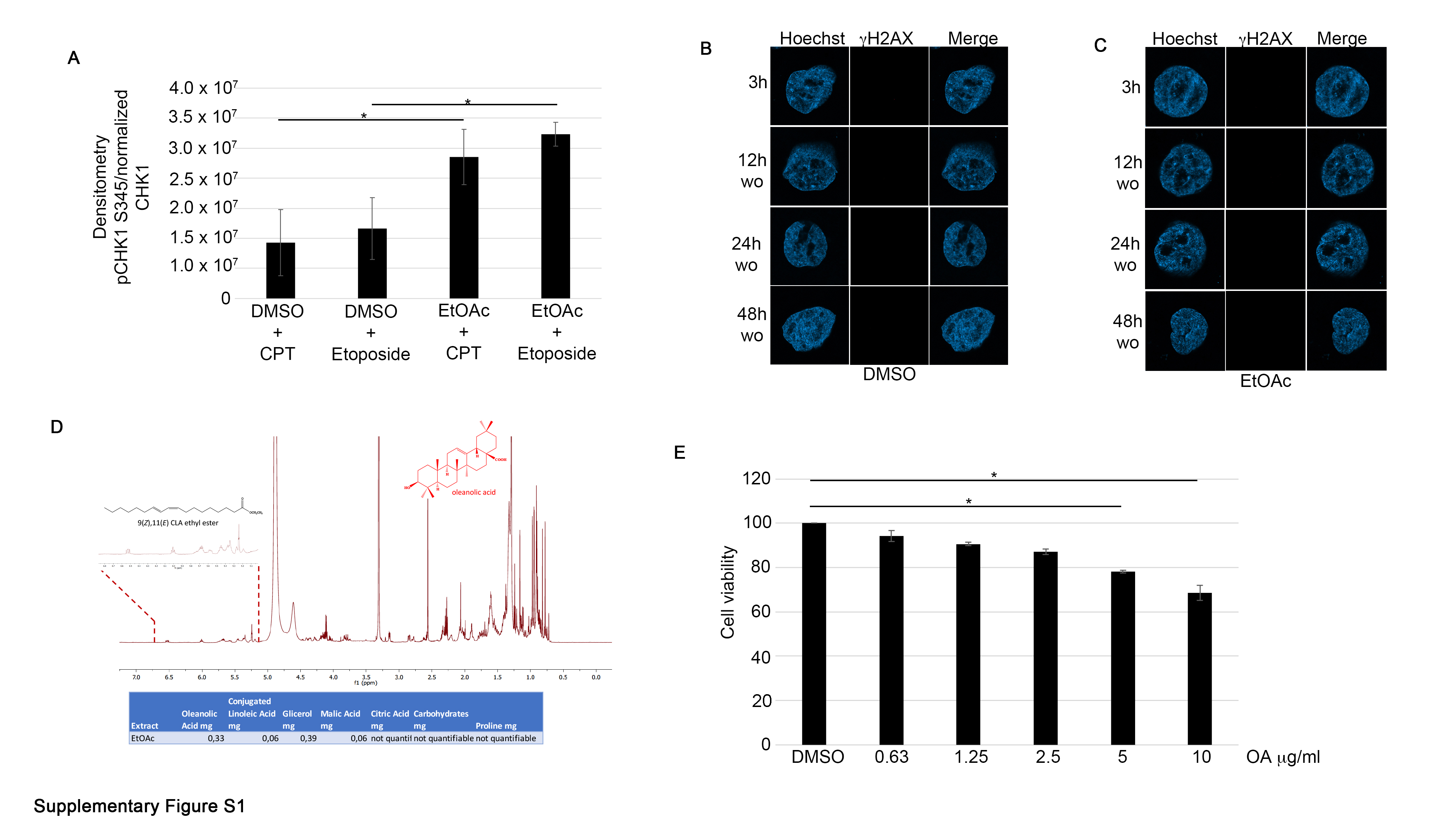

Supplement: Supplementary file 1 [file ijms-25-13475-s001.zip › Supplementary Figure S1.jpg]

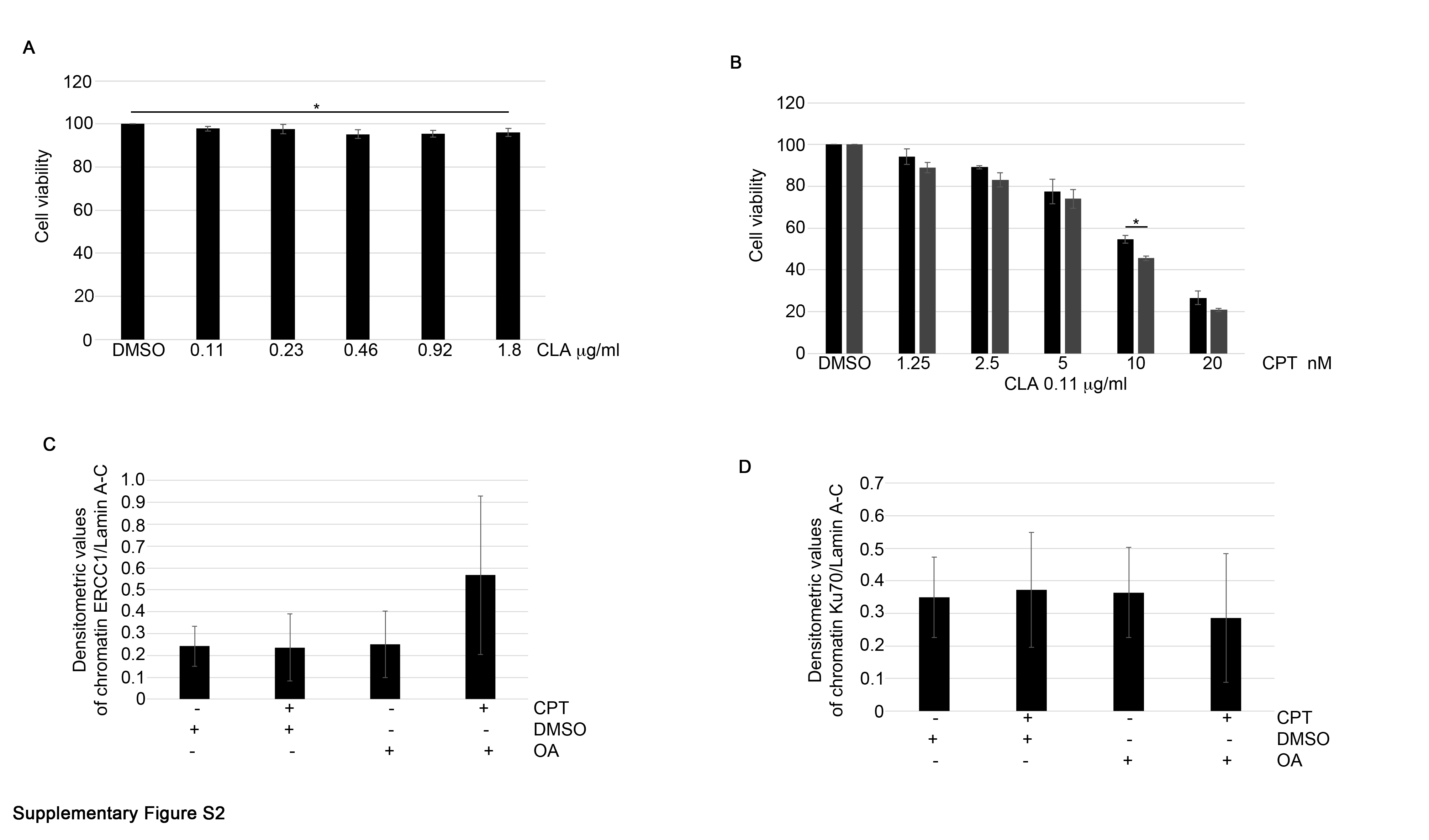

Supplement: Supplementary file 1 [file ijms-25-13475-s001.zip › Supplementary Figure S2.jpg]
